# Targeted B-domain deletion restores *F8* function in human endothelial cells and mice

**DOI:** 10.1038/s41392-022-01016-9

**Published:** 2022-06-20

**Authors:** Zhiqing Hu, Zhuo Li, Yong Wu, Junya Zhao, Lingqian Wu, Miaojin Zhou, Desheng Liang

**Affiliations:** Center for Medical Genetics, School of Life Sciences, Central South University, Changsha, Hunan 410078 China

**Keywords:** Medical genetics, Stem-cell differentiation, Pluripotent stem cells

**Dear Editor**,

Hemophilia A (HA) is the most common genetic bleeding disorders and affects 1 in 5000 male births.^1^ HA patients suffer spontaneous soft-tissue and joint bleeding or life-threatening intracranial hemorrhage. The main clinical treatment for severe HA patients is life-long maintenance of protein replacement therapy, which increase the risks of infection and induce FVIII inhibitors. Gene therapy is expected to cure HA, however, the current AAV-based gene addition strategy is limited by its loading capacity, immunogenicity of capsids, and the risk of insertional mutagenesis.^2^ Therefore, the aim of this study is to overcome these limitations by a combination of gene editing and ex vivo strategy for HA gene therapy.

Genetically, HA is caused by mutations in *F8* encoding coagulation factor VIII (FVIII), which comprises three A domains, one B domain (~44% of FVIII), and two C domains. It is reported that 15–26% of severe HA patients harbor mutations involving the sequences coding FVIII B domain (*F8*-B), second only to the intron 22-inversion mutation (45%). Notably, the FVIII that ultimately exerts coagulation function does not contain the B domain. In recent decades, a version of FVIII lacking most of the B domain (BDD-FVIII) was developed, and exogenous BDD-FVIII or BDD-*F8* subsequently appeared to be safe and effective in preclinical studies or clinical management of HA patients.^3^ FVIII harbors 19 N-linked glycosylation sites within B domain. Partial B-domain-deleted FVIII, which retains several N-linked glycosylation sites, secretes more efficiently due to increased endoplasmic reticulum-to-Golgi transport.^4^ However, it remains unclear whether endogenous FVIII with either partial or entire *F8*-B deletion retains coagulation function. Recently, we discovered that reframed *F8* within 54-bp deletion in *F8*-B could express functional FVIII, and hence hypothesized that in situ genetic manipulations of *F8* to create an entire *F8*-B deletion might represent a therapeutic strategy for all HA patients with mutations in *F8*-B. Here we evaluated this strategy in HA patient-derived induced pluripotent stem cells (HA-iPSCs) and normal human iPSCs (N-iPSCs) using clustered regularly interspaced short palindromic repeats-associated protein-9 nuclease (CRISPR/Cas9) (Fig. 1a).

**Fig. 1:**
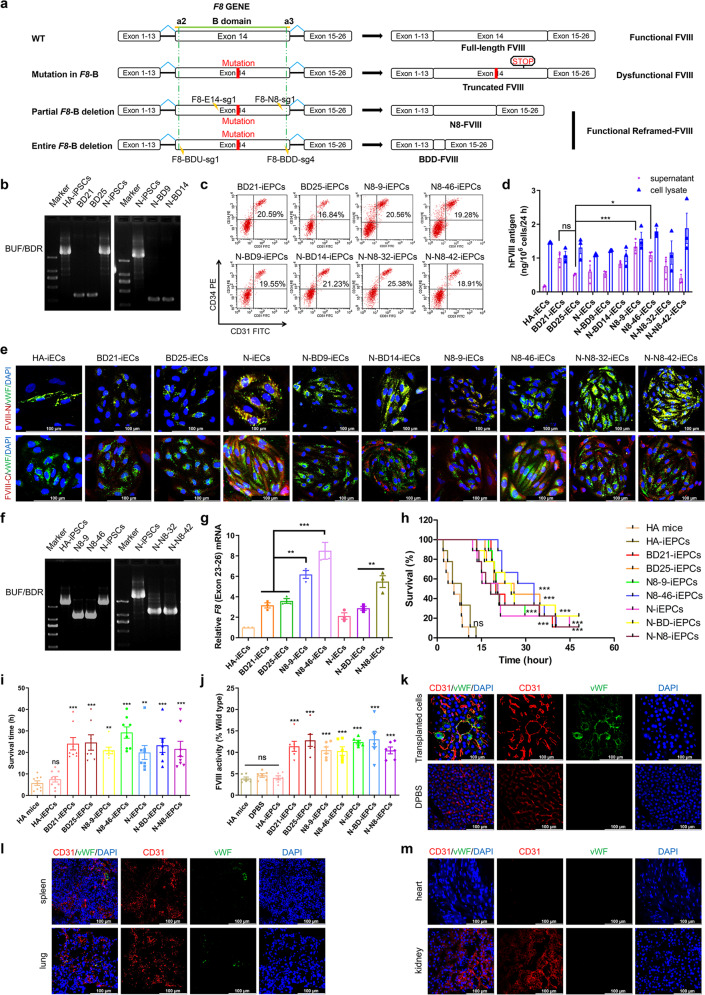
Functional restoration of FVIII in human ECs and mice via targeted entire or partial B-domain deletion of the endogenous *F8* gene. **a** Schematic representation of generation of B-domain targeted deletion and 8 N-linked glycosylation sites retained in B domain of the endogenous *F8* gene. **b** PCR screening of BD-iPSCs using the primers BUF/BDR. Sizes of the PCR products: B-domain deletion, 341 bp; N-iPSCs, 3023 bp; HA-iPSCs, 3019 bp. **c** EPCs differentiation efficiency was determined by FACS analysis of CD31 and CD34 expression. **d** ELISA analysis of the FVIII antigen in iPSC-derived iECs. Data represent the mean ± SEM (*n* = 3 independent cultures). n.s., not significant compared with BD25-iECs. *p < 0.05, ***p < 0.001, vs. the BD25-iECs group. **e** Immunofluorescence staining of FVIII (red) and vWF (green) in iECs, DAPI was used for nuclear staining. FVIII-N, F8 protein N-terminus. FVIII-C, F8 protein C-terminus. **f** PCR screening of N8-iPSCs. The sizes of the PCR products using primers BUF/BDR for the N8-iPSCs was 1160 bp, N-iPSCs was 3023 bp, and HA-iPSCs was 3019 bp. **g** qRT-PCR analysis of *F8* expression in iECs using primers targeting exons 23 and 26 (E23-26), with *GAPDH* used as a loading control. **h** Proportions of surviving mice after tail-clip challenge. HA mice, hemophilia A mice (*n* = 9); HA mice transplanted with HA-iEPCs (*n* = 9), BD21-iEPCs (*n* = 9), BD25-iEPCs (*n* = 9), N8-9-iEPCs (*n* = 9), N8-46-iEPCs (*n* = 9), N-iEPCs (*n* = 9), N-BD-iEPCs (*n* = 9), and N-N8-iEPCs (*n* = 9), respectively. n.s., not significant compared with HA mice. ***p < 0.001, vs. the HA-iEPCs group (log-rank test). **i** Average survival time of mice. Note that the mice that survived the challenge were excluded in this analysis. Data represent the mean ± SEM. n.s., not significant compared with HA mice. ***p < 0.001, **p < 0.01, vs. the HA-iEPC group. **j** Relative FVIII activity detected at 2-week post-transplantation in HA mice, HA mice without transplantation (*n* = 6); HA mice transplanted with DPBS (*n* = 6), HA-iEPCs (*n* = 6), BD21-iEPCs (*n* = 6), BD25-iEPCs (*n* = 6), N8-9-iEPCs (*n* = 6), N8-46-iEPCs (*n* = 6), N-iEPCs (*n* = 6), N-BD-iEPCs (*n* = 6), and N-N8-iEPCs (*n* = 6), respectively. Data represent the mean ± SEM. ***p < 0.001, vs. the HA-iEPC group. **k** Liver tissue sections of HA mice transplanted with BD-iEPCs were analyzed using immunofluorescence with anti-human vWF (green) and CD31 (red) antibodies. No signal was found in the DPBS group. DAPI was used for nuclear staining. **l** Tissue sections of other organs from mice transplanted with BD-iEPCs were analyzed by immunofluorescence. Cells positive for anti-human vWF staining were observed in the spleen and lung. CD31 (red), vWF (green), with DAPI used for nuclear staining. **m** No signal was found in the heart or kidney. CD31 (red), vWF (green), with DAPI used for nuclear staining

HA-iPSCs were previously generated from urine cells of an HA patient (c.3167del CTGA).^5^ To rule out re-cutting of the fused sequences (Ser743 fused to Gln1638) by CRISPR/Cas9, we retained the coding sequences for Gln744 and Asn745 to ensure the fusion of Asn745 to Pro1640 (Supplementary Fig. S1a). The dual single-guide RNAs (sgRNAs) F8-BDU-sg1 and F8-BDD-sg4 were designed with verification of the cleavage activity (Supplementary Fig. S1b). Plasmids expressing the CRISPR/Cas9 complex and sgRNAs along with the corresponding ssODN template were nucleofected into the HA-iPSC and N-iPSC lines. We achieved high targeting efficiency of 4.17% without any screening (Fig. 1b and Supplementary Fig. S1c). The stable BDD-*F8* clones maintained pluripotency and a normal karyotype (Supplementary Fig. S1d–f). No off-target indels were observed (Supplementary Fig. S2). The reframed *F8* transcript of the BD-iPSCs was detected (Supplementary Fig. S3a); however, almost no FVIII was detected in supernatant from iPSC culture (Supplementary Fig. S3b). Western blot revealed almost undetectable levels of lectin mannose-binding 1 (LMAN1), which reportedly mediates FVIII secretion (Supplementary Fig. S3c).

Given that ECs are the main cell type secreting FVIII, we differentiated BD-iPSCs into EPCs/ECs and evaluated FVIII expression in BD-iPSC-derived EPCs (BD-iEPCs) and ECs (BD-iECs). The differentiation efficiencies ranged from 16 to 26% (Fig. 1c). Pure iEPCs expressing CD31 and CD144 and mature ECs expressing von Willebrand factor (vWF) were obtained (Supplementary Fig. S3d). *F8* transcription in BD-iECs was detected (Supplementary Fig. S3e). The secreted FVIII was 1.00 ng/10^6^ cells and 0.52 ng/10^6^ cells from BD21-iECs and BD25-iECs respectively, both of which were higher than that from HA-iECs (0.17 ng/10^6^ cells) and close to that from N-iECs (0.60 ng/10^6^ cells) (Fig. 1d). We detected LMAN1 in ECs, possibly explaining why FVIII secreted from iECs but not from iPSCs (Supplementary Fig. S3c). Importantly, immunostaining by both N-terminal and C-terminal FVIII antibody revealed restoration of FVIII expression in BD-iECs, whereas the truncated FVIII was detected in HA-iECs by a N-terminal FVIII antibody (Fig. 1e). These results demonstrated an expression and secretion of endogenous BDD-FVIII in BD-iECs.

To determine the expression efficiency of endogenous N8-FVIII, we constructed an in situ partial B-domain-deleted N8-FVIII variant harboring 271 amino acids at the N-terminus (Fig. 1f and Supplementary Fig. S4a). The iPSCs harboring the N8-FVIII variant were obtained with an efficiency of 14.58% (Supplementary Fig. S4b–d). N8-9-iPSCs and N8-46-iPSCs clones that expressed pluripotent genes, maintained a normal karyotype, and transcribed reframed N8-FVIII (Supplementary Fig. S4e–g) were selected for further experiments. Almost no FVIII was detected in culture supernatant of iPSCs (Supplementary Fig. S4h, i). Sanger sequencing revealed no indels at the putative off-target sites (Supplementary Fig. S5). N8-iPSCs were differentiated to EPCs (N8-iEPCs) and ECs (N8-iECs) (Fig. 1c and Supplementary Fig. S6a) that were characterized by angiogenesis and Dil-acetylated-low-density-lipoprotein endocytosis (Supplementary Fig. S6b, c). The reframed N8-FVIII transcripts were detected in N8-iECs (Supplementary Fig. S6d), which were eightfold higher than those in HA-iECs and threefold higher than those in BD-iECs (Fig. 1g), although well lower than that in primary liver sinusoidal endothelial cells (LSECs) with highest FVIII production capacity described previously. FVIII levels in supernatant of N8-9-iECs and N8-46-iECs were significantly higher than that in BD-iECs (Fig. 1d). Meanwhile, FVIII expression in N8-iECs was confirmed by immunostaining (Fig. 1e), and LMAN1 was detected in ECs (Supplementary Fig. S6e).

To validate the therapeutic effects of endogenous BD-FVIII and N8-FVIII, we transplanted HA-iEPCs, reframed iEPCs, and N-iEPCs into HA mice via retro-orbital vein injection. By 2 weeks post-infusion, HA mice were subjected to a tail-clip challenge and plasma FVIII activity assays. The average survival time of HA mice transplanted with BD-iEPCs and N8-iEPCs (24.62 h and 29.25 h, respectively) were significantly longer than those of untreated mice (5.73 h) (Fig. 1h, i). Notably, three of the 18 HA mice transplanted with BD-iEPCs and 3 of the 18 HA mice with N8-iEPCs were alive 48 h after tail-clip challenge (the experiment endpoint). Furthermore, we observed higher plasma FVIII activities in HA mice transplanted with BD-iEPCs and N8-iEPCs (12.79% and 10.49%, respectively) than that in HA mice (3.88%) (Fig. 1j). These results indicated that endogenous FVIII with partial or entire deletion of *F8*-B could exert coagulation function and systematic infusion of the reframed iEPCs could rescue FVIII deficiency in HA mice, firstly demonstrating in vitro and in vivo functionality of FVIII which is encoded by endogenous *F8* with deletion of the entire B domain. Using immunostaining of anti-human vWF antibody, we identified positive cells in the livers of HA mice transplanted with iEPCs but not in mice injected with Dulbecco’s phosphate-buffered saline (DPBS) (Fig. 1k), and positive signals were observed in the lungs and spleens of iEPC-transplanted mice but not in the heart or kidney (Fig. 1l, m). Additionally, no obvious damage to the liver or kidney was observed within the 2-week observation period (Supplementary Fig. S6f–h).

In summary, we achieved an efficient targeted *F8*-B deletion in HA-iPSCs and the derived iECs could express functional FVIII. Transplantation with the *F8*-B-deleted iEPCs could restore FVIII function and rescue the bleeding phenotype in HA mice. These findings provide a proof of concept for the production of the functional FVIII from endogenous *F8* with targeted B-domain deletion and preclinical validation of an autologous stem cell gene therapy for HA.

## Supplementary information

Supplementary Materials

## Data Availability

All data relevant to this work are included in this paper and Supplementary Information.
