# Supplementary material for: Targeted B-domain deletion restores F8 function in human endothelial cells and mice
Source: Signal Transduct Target Ther. 2022 Jun 20;7:189. doi: 10.1038/s41392-022-01016-9 (PMC9207027; doi:10.1038/s41392-022-01016-9)
Supplement: Supplementary file 1 — Supplementary Materials [file 41392_2022_1016_MOESM1_ESM.docx]

Supplementary Materials for

Targeted B-domain deletion restores F8 function in human endothelial cells and mice

Zhiqing Hu,^1^ Zhuo Li,^1^ Yong Wu,^1^ Junya Zhao,^1^ Lingqian Wu,^1^ Miaojin Zhou,^1,*^ Desheng Liang^1,*^

1Center for Medical Genetics, School of Life Sciences, Central South University, Changsha, Hunan, 410078, China

Correspondence to: Desheng Liang ([liangdesheng@sklmg.edu.cn](mailto:liangdesheng@sklmg.edu.cn)) and

Miaojin Zhou ([zhoumiaojin@sklmg.edu.cn](mailto:zhoumiaojin@sklmg.edu.cn))

**This PDF file includes:**

Materials and Methods

Figures. S1 to S6

Tables S1 to S4

**MATERIALS AND METHODS**

**RGEN design and plasmid construction**

All sgRNAs were designed using the online tool CRISPOR (<http://crispor.tefor.net/>),^1^ with short guide RNA oligos synthesized by Sangon Biotech. The RGEN plasmid pX330 (pX330-U6-Chimeric_BB-CBh-hSpCas9) was a gift from Feng Zhang (Addgene plasmid #42230; http://n2t.net/addgene:42230, Watertown, MA, USA).^2^ pX330 was digested with *BbsI* (New England Biolabs #R3539, Ipswich, MA, USA), gel purified, and ligated along with the annealed complementary sgRNA oligos using T4 DNA ligase (Thermo Fisher Scientific #EL0011, Waltham, MA, USA). Plasmid constructs were confirmed by Sanger sequencing. To detect RGEN activity, HEK293T cells were transfected with CRISPR/Cas9 plasmid using Lipofectamine 2000 (Invitrogen #11668-019, Carlsbad, CA, USA) according to manufacturer instructions with the transfection efficiency indicated by the GFP plasmid transfection group. When the transfection efficiency reaches more than 90%, the follow-up detection was performed. Genomic DNA of transfected cells were extracted 48 h after transfection, and the region encompassing the targeted locus was PCR amplified, the PCR products were ligated with T vector and transformed into competent *E. coli*. Over 28 clones were picked and sequenced by Sanger sequencing. F8-E14-sg1 used in this study was described previously.^3^

**Gene targeting**

HA-iPSCs cultured on Matrigel (Corning #354277, NY, USA) were dissociated with TrypLE Select (Life Technologies #A1217701, Grand Island, NY, USA) at 37 °C for 3 min. Subsequently, single cells were collected, counted, and resuspended with 100 µL of reagent from the human stem cell Nucleofector kit 2 (Lonza #VPH-5022, Alpharetta, GA, USA) for nucleofection using Nucleofector II (Lonza) set at program B016. For the BD group, 2.5 µg of each CRISPR/Cas9 plasmid was used along with 50 pmol of ssODN synthesized by Sangon Biotech to transfect 1×10^6^ cells (HA-iPSCs and N-iPSCs). For 1×10^6^ cells, 2.5 µg of each CRISPR/Cas9 plasmid used without ssODN for generation of partial B-domain-deletion clones (N8-iPSCs harboring an N-terminus 271 amino acids containing eight putative asparagine-linked glycosylation sites). The transfected cells were maintained on Matrigel-coated wells in mTeSR1 (STEMCELL Technologies # 85850, Vancouver, BC, Canada) containing 10 µM Y27632 (STEMCELL Technologies #72304). After 2 days, the cells were dissociated into single cell, counted, and then 5000 cells were seeded onto mouse embryonic fibroblast-feeders in ES medium containing 10 µM Y27632 in a 100-mm culture dish. Approximately 12 days later, clones were mechanically selected and expanded, and the modified targeted genome sequences were identified by PCR and Sanger sequencing. For both the BD and N8 groups, PCR was performed using two pairs of primers: F8-E14-F, 5'-GGTGGACCTCTGAGCTTGAGTGA-3' and F8-E14-R, 5'-GCATCTTAAAGAACGACATATCTGGAT-3'; and F8-BU-F, 5'-CATGACCGCCTTACTGAAGGT-3' and F8-BD-R, 5'-AATCCCAGAGCCTCTCCACT-3'. For the BD group, the 341-bp product amplified by F8-BU-F and F8-BD-R was sequenced, and for the N8 group, the 1160-bp product amplified by F8-BU-F and F8-BD-R was sequenced by Sanger sequencing.

**Analysis of potential off-target sites**

To examine the present of off-target sites in gene-edited clones, we used Sanger sequencing for HA-iPSCs, BD-iPSCs, and N8-iPSCs. The potential off-target sites for mismatches with CRISPR/Cas9 on-target sites of up to three nucleotides were searched using CHOPCHOP ([http://chopchop.cbu.uib.no/)](http://chopchop.cbu.uib.no/).).^4^ Genomic DNA from HA-iPSCs and BD-iPSCs was PCR amplified targeting eight potential off-target sites for F8-BDU-sg1 and three potential off-target sites for F8-BDD-sg4 (Supplementary information, Table S2), followed by Sanger sequencing of the PCR products. DNA from HA-iPSCs and N8-iPSCs was PCR amplified for five potential off-target sites for F8-E14-sg1 and ten potential off-target sites for F8-N8-sg1 (Supplementary information, Table S3), and the PCR products were sequenced. Indels were used to evaluate off-target effects in genetically edited iPSCs. Primer sequences are shown in Supplementary information, Table S4.

**iPSCs characterization**

Immunofluorescence staining of iPSC surface markers was performed. Briefly, cells were fixed with 4% paraformaldehyde for 15 min, permeabilized with 0.1% Triton-X100 in DPBS (Thermo Fisher Scientific #C14190500BT) for 15 min and blocked in 5% bovine serum albumin (BSA, Geneview #FA016, St. Galveston, TX, USA) for 30 min, followed by incubation with primary antibody diluted 1:100 in blocking buffer (OCT4 (Abcam #ab181557, Cambridge, UK), NANOG (Abcam #ab109250), stage-specific embryonic antigen (SSEA)-1 and SSEA-4 (Merck Millipore #SCR001, Billerica, MA, USA)) at 25 °C for 1 h. After washing thoroughly with DPBS, cells were blocked with 5% BSA for 30 min and incubated with secondary antibodies for 1 h. 4’, 6’-diamidino-2-phenylindole (DAPI) (Thermo Fisher Scientific #D1306) was used for nuclear staining. Stained cells were photographed with a ﬂuorescence microscope (Leica DM IRB, Wetzlar, Germany).

**Karyotype analysis of iPSCs**

For karyotype analysis, G-banding analysis of chromosomes from each iPSC line was performed. iPSCs were treated with 0.1 μg/mL colcemid (Sigma-Aldrich #D7385, St. Louis, MO, USA) for 4 h, trypsinized, pelleted, and then treated with 0.075 M KCl for 10 min at 37 °C. After fixation with Carnoy fixative, metaphase chromosome spreads were prepared via the air-drying method. The G-banded analyzed chromosomes were stained with Giemsa (Sigma-Aldrich #48900) and analyzed under the microscope.

**Differentiation into EPCs from human iPSCs**

Derivation of EPCs from human iPSCs was performed using small-molecule-mediated activation of WNT signaling, as previously described.^5^ Briefly, iPS cells were detached with Accutase (Thermo Fisher Scientific #A1110501), plated on Matrigel-coated wells at 50,000 cells/cm^2^ in mTeSR1 containing 10 µM Y27632, and cultured for 3 days, after which they were cultured in LaSR basal medium (Advanced/F12, Thermo Fisher Scientific #12634010; 2.5 mM GlutaMAX, Thermo Fisher Scientific #35050061 and 60 mg/mL ascorbic acid, Sigma-Aldrich #A4544-25G) supplemented with 6 µM CHIR99021 (Selleck Chemicals #S2924, Houston, TX, USA) for 2 days. The cells were then cultured in LaSR basal medium for an additional 3 days, followed by characterization via flow cytometry and immunofluorescent staining. The EPCs were purified with a CD31 microbead kit (Miltenyi Biotec #130-091-935, Auburn, CA, USA) according to manufacturer instructions and cultured in Matrigel-coated wells in EGM-2 medium (Lonza #CC3202) to allow maturation.

**Characterization of EPCs and ECs**

Immunofluorescence staining of EC surface markers was performed using the same protocol as that described for iPSC characterization. The primary antibodies used were anti-CD31 (Sigma-Aldrich #P8590), anti-vWF (Abcam #ab109250), anti-FVIII N-terminus antibody (Santa Cruz Biotechnology #sc27649, Dallas, TX, USA), and anti-FVIII C-terminus antibody (Sekisui Diagnostica #ESH-8, Greenwich, CT, USA). DAPI was used for nuclear staining. Stained cells were photographed with a ﬂuorescence microscope. For flow cytometric analysis, differentiated cells were harvested with Accutase, resuspended in DPBS, and divided into 1×10^6^ cells/100 µL aliquots. PE-conjugated monoclonal antibodies (mAbs) against human CD34 (BD biosciences #550761, Franklin Lakes, NJ, USA) and FITC-conjugated mAbs against CD31 (BD biosciences #560984) were added to the aliquots, respectively. After 30 min incubation at room temperature in the dark, cells were washed with 1 mL DPBS, resuspended in 500 µL DPBS, and analyzed using a FACS Calibur flow cytometer (BD Biosciences).

**Vascular tube formation and acetylated low-density lipoprotein (AcLDL) uptake**

To evaluate tube formation, 5×10^4^ iECs were cultured in Matrigel-coated 48-well plates for 16 h using EGM-2 medium (Lonza #CC-3202). For the AcLDL-uptake assay, when the ECs reach 30-40% confluency, the cells were rinsed with DPBS 3 times, then incubated with 2.4 µg/mL Dil-AcLDL (Thermo Fisher Scientific #L3484) diluted in EGM-2 medium for 4 h at 37 °C, washed with DPBS 3 times, then observed via fluorescence microscopy and red fluorescence can be observed in ECs.

**RT-PCR**

Total RNA was isolated using TRIzol reagent (Sigma-Aldrich #T9424) and digested with DNase for 30 min to remove potential residual DNA. RNA was then reverse transcribed using HiScript II 1st Strand cDNA Synthesis Kit (Vazyme #R212, Nanjing, China). For the BD group, two pairs of primers were used to detect the *F8* transcripts. One pair of primers was based on exons 13 and 15 and contained the deleted fragment, and the other was based on exons 23 and 26. For the N8 group, three pairs of primers were used to detect the *F8* transcripts. One pair of primers was based on exons 14 and 15, and one pair of primers was based on exons 19 and 23, and the other was based on exons 23 and 26. Glyceraldehyde-3-phosphate dehydrogenase (GAPDH) was used as an endogenous control. Primer sequences are shown in Supplementary information, Table S1.

**Quantitative reverse transcription PCR (qRT-PCR) analysis**

Total RNA was isolated with TRIzol reagent and treated with DNase for 30 min to eliminate any residual DNA. qRT-PCR was performed with a HiScript II One Step qRT-PCR SYBR Green Kit (Vazyme #Q711) according to the manufacturer’s instructions in a Bio-Rad CFX96 Touch q-PCR system. The *GAPDH* gene was ampliﬁed as an endogenous control. All the primers are shown in Supplementary information, Table S1.

**FVIII ELISA**

Culture supernatants were harvested from 12-well plates after medium replacement for 24 h. The cells were detached with Accutase, counted, resuspended in 500 µL of sample diluent for ELISA (Cedarlane #CL20035K, Burlington, ON, Canada) and lysed via three freeze-thaw cycles. All samples were collected in triplicate. ELISA was performed with paired antibodies for ELISA-Factor VIII:C (Cedarlane #CL20035K) according to manufacturer instructions. The standard curves were constructed depending on detecting of serial dilutions of normal pooled plasma, with a correlation coefﬁcient (R^2^) of at least 0.990 using a semilog ﬁt. The concentration of each sample was calculated according to the standard curve and the secretion intensity was showed as secretion amount of 1 million cells.

**Western blot analysis**

iPSCs and derived ECs were lysed with lysis buffer containing 1% protease-inhibitor cocktail for 5 min on ice, followed by collection and sonication. Lysates were heated at 95 °C for 10 min, quantified using a BCA protein assay (Thermo Fisher Scientific #23227) and stored at -80 °C. Protein samples (20 μg) per group mixed with loading buffer were subjected to 10% polyacrylamide gel electrophoresis, followed by transfer onto polyvinylidene fluoride membranes. After blocking with 5% skimmed milk in 0.1% Tris-buffered saline with Tween-20, the membranes were incubated with mouse anti-LMAN1 (1:1000, Abcam #ab118407) and rabbit anti-β-Actin (1:10000, Sigma-Aldrich #A2066) overnight at 4 °C. After washing with 0.1% Tris-buffered saline with Tween-20, the membranes were incubated with anti-mouse and anti-rabbit horseradish peroxidase-conjugated secondary antibodies (1:10000, Abcam #ab6728 and #ab6721) for 1 h at room temperature, followed by signals processing by enhanced chemiluminescence. A pre-stained protein ladder was used to estimate the apparent molecular weight (Thermo Fisher Scientific #26619).

**iEPCs transplantation into HA mice**

HA mice (strain: B6; 129S-F8 tm1Kaz/J; 6-weeks old, stock no.004424, Jackson Laboratory) were obtained from Jackson Laboratory (Bar Harbor, ME, USA) and used for *in vivo* functional assays. The care and use of the animals complied with the guidelines of the Ethics Committee of the School of Life Sciences of Central South University. All animal experiments were approved by the Institutional Animal Care and Use Committee of School of Life Sciences of Central South University.

For *in vivo* transplantation, C57BL/6 mice (6-weeks old) were used as wild-type controls. The number of males and females in each group was same. After HA mice were anesthetized with avertin (2,2,2-Tribromoethanol, Sigma-Aldrich #T48402-5G and 2-Methyl-2-butanol, Sigma-Aldrich #152463-250ML) via intraperitoneal injection, 2×10^6^ iEPCs derived from HA-iPSCs, BD-iPSCs, N8-iPSCs, or N-iPSCs were infused into each mouse via orbital vein injection. After transplantation, FK506 (Selleck Chemicals #S5003, 1 mg/kg in 100 µL corn oil, Sigma-Aldrich #C8267) was intraperitoneally injected once every other day. At 2-weeks post-transplantation, treatment efficacy was evaluated by tail clip challenge.^6^ Briefly, the distal part of the mouse tail with a diameter of 1.5 mm was sheared and allowed to bleed for 5 min after the mice were anesthetized. After applying firm pressure to the tail for 1 min, the survival time was recorded and monitored until 48 h after the tail-clip. Tissues (heart, liver, spleen, lung and kidney) from cell-transplanted mice were harvested in 4% paraformaldehyde and analyzed using tissue immunofluorescence. Briefly, the tissues were frozen sliced and baked at 75 °C, then permeabilized with 0.1% Triton-X100 in DPBS for 17 min and blocked in 5% BSA for 30 min, then incubated with primary antibody diluted 1:100 in blocking buffer (anti-CD31, Sigma-Aldrich #P8590; anti-vWF, Abcam #ab109250) at 4 °C overnight, washed with DPBS, followed by blocking with 5% BSA for 30 min and incubated with secondary antibodies for 1 h. DAPI was used for nuclear staining.

Blood samples were collected from the eyes of treated mice after anesthetization in order to isolate plasma for FVIII activity assays and isolate serum for detection of markers of liver injury and kidney injury. For the FVIII activity assay, coagulation factor VIII-deﬁcient plasma (Siemens Healthcare Diagnostics, Marburg, Germany) and a Destiny Max hemostasis analyzer (Tcoag, Lemgo, Germany) were used to test the activated partial thromboplastin time (aPTT). All operations were performed according to the manufacturer’s instructions.

**Statistical analysis**

Data were analyzed with GraphPad Prism software (v.5.01; GraphPad Software, La Jolla, CA, USA). One-way analysis of variance (ANOVA) was performed to compare data among three or more groups, and the log-rank test was used for survival curve analysis. All values are presented as the mean ± standard error of the mean (SEM).

**Reference**

1. Concordet, J.P. and M. Haeussler. CRISPOR: intuitive guide selection for CRISPR/Cas9 genome editing experiments and screens. *Nucleic Acids Res*. **46**, W242-W245 (2018).

2. Cong, L., et al. Multiplex genome engineering using CRISPR/Cas systems. *Science*. **339**, 819-23 (2013).

3. Hu, Z., et al. ssODN-Mediated In-Frame Deletion with CRISPR/Cas9 Restores FVIII Function in Hemophilia A-Patient-Derived iPSCs and ECs. *Mol Ther Nucleic Acids*. **17**, 198-209 (2019).

4. Labun, K., et al. CHOPCHOP v3: expanding the CRISPR web toolbox beyond genome editing. *Nucleic Acids Res*. **47**, W171-W174 (2019).

5. Lian, X., et al. Efficient differentiation of human pluripotent stem cells to endothelial progenitors via small-molecule activation of WNT signaling. *Stem Cell Reports*. **3**, 804-16 (2014).

6. Park, C.Y., et al. Functional Correction of Large Factor VIII Gene Chromosomal Inversions in Hemophilia A Patient-Derived iPSCs Using CRISPR-Cas9. *Cell Stem Cell*. **17**, 213-20 (2015).

Figures. S1 to S6

Figure. S1.


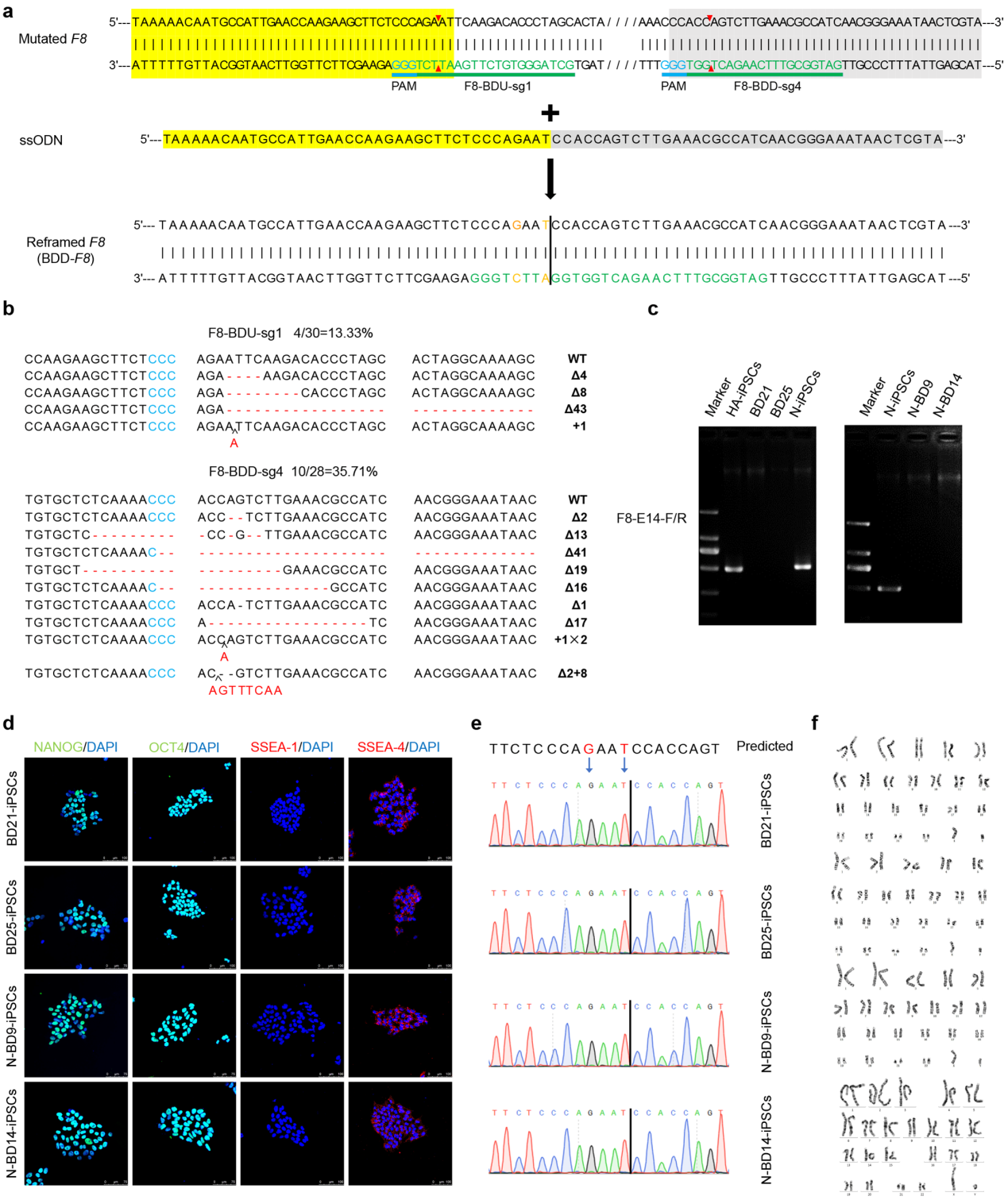


**Figure S1. CRISPR/Cas9 and ssODN-mediated generation of BD-iPSCs.**

**a** Schematic representation of CRISPR/Cas9-mediated B-domain targeted deletion. The design of two sgRNAs for the *F8*-B-coding sequences in *F8* exon 14. Yellow-shaded bases are left homologous sequences, and gray-shaded bases are right homologous sequences. Green letters represent the sgRNA sequences, and blue letters represent the protospacer-adjacent motif (PAM). The red arrow shows the CRISPR/Cas9 cut sites. Reframed *F8* (BDD-*F8*) shows a B-domain deletion with the SQ sequence and correction of the reading frame of frameshift mutations in this region. The vertical line indicates the upstream and downstream connections. The orange letters are the two synonymous mutations. **b** Sanger sequencing of the indels introduced by F8-BDU-sg1 or F8-BDD-sg4 in HEK-293T cells. WT, wild type; △, deletion; +, insertion; ×, times. **c** PCR screening of BD-iPSCs using primers F8-E14-F/R. No PCR product was obtained for the BD-iPSCs. PCR products sizes: N-iPSCs, 502 bp; and HA-iPSCs, 498 bp. **d** Immunofluorescence indicated that BD-iPSCs expressed the markers NANOG, OCT4, SSEA-4 but not SSEA-1. 4’,6’-diamidino-2-phenylindole (DAPI) was used for nuclear staining. **e** The PCR products for BD-iPSCs using primer BUF/BDR were sequenced by Sanger sequencing. **f** Karyotype of BD-iPSCs.


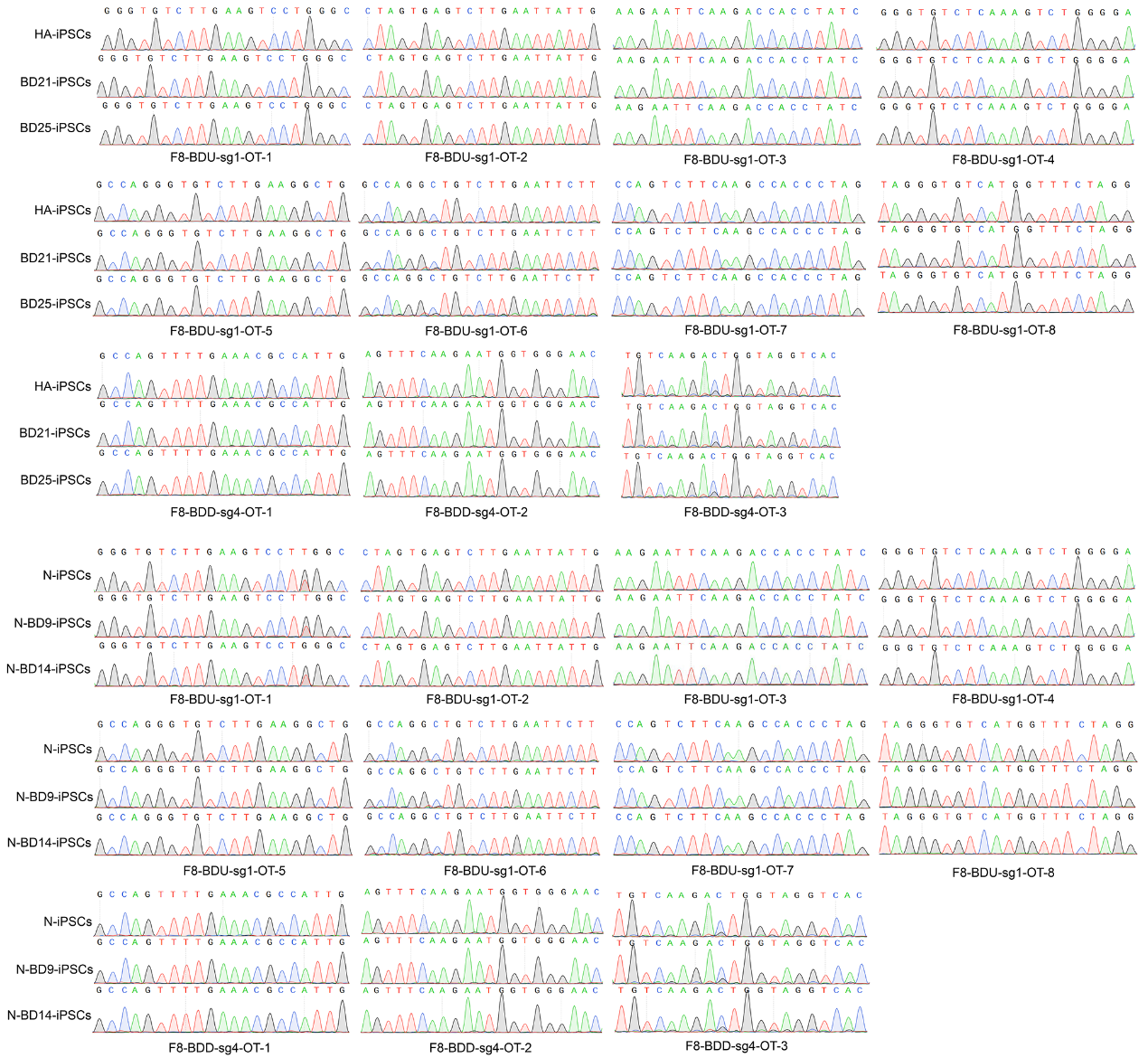
Figure. S2.

**Figure S2. Sanger sequencing of potential off-target sites in BD-iPSCs using F8-BDU-sg1 and F8-BDD-sg4**.

We detected the initial patient cells (HA-iPSCs) and the B domain deleted-clones (BD21-iPSCs and BD25-iPSCs). And also, we analyzed the N-iPSCs and the B domain deleted-clones (N-BD9-iPSCs and N-BD14-iPSCs). No indels were found at the sites.


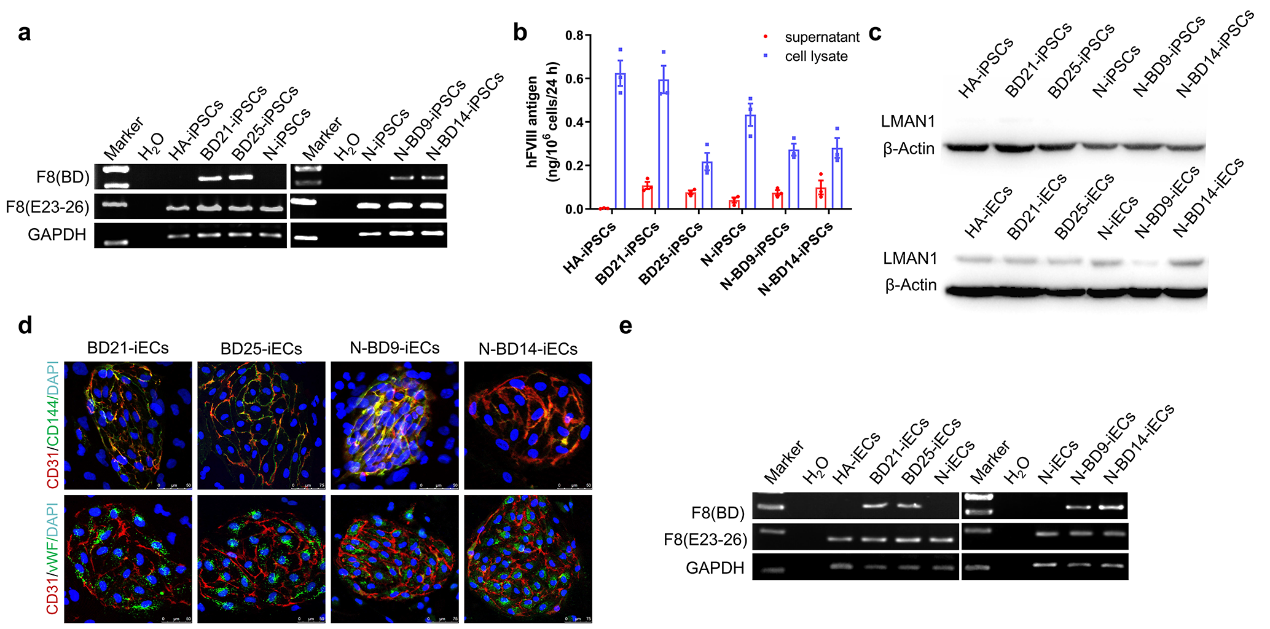
Figure. S3.

**Figure S3. Restoration of FVIII expression in BD-iPSC-derived EPCs (BD-iEPCs).**

**a** RT-PCR analysis of *F8* transcription in BD-iPSCs. *GAPDH* was used as a loading control. H_2_O was used as a blank control. **b** ELISA detection of FVIII antigen in iPSCs. Data represent the mean ± SEM (n = 3 independent cultures). **c** LMAN1 expression in iPSCs and ECs according to western blot, with β-Actin used as the internal reference. **d** Immunofluorescence staining of CD31 (red), CD144 (green), and vWF (green) in BD-iECs. **e** *F8* expression in iECs according to RT-PCR. F8(BD) using primers targeting exons 13 and 15, F8(E23-26) using primers targeting exons 23 and 26. *GAPDH* was used as a loading control.

Figure. S4.


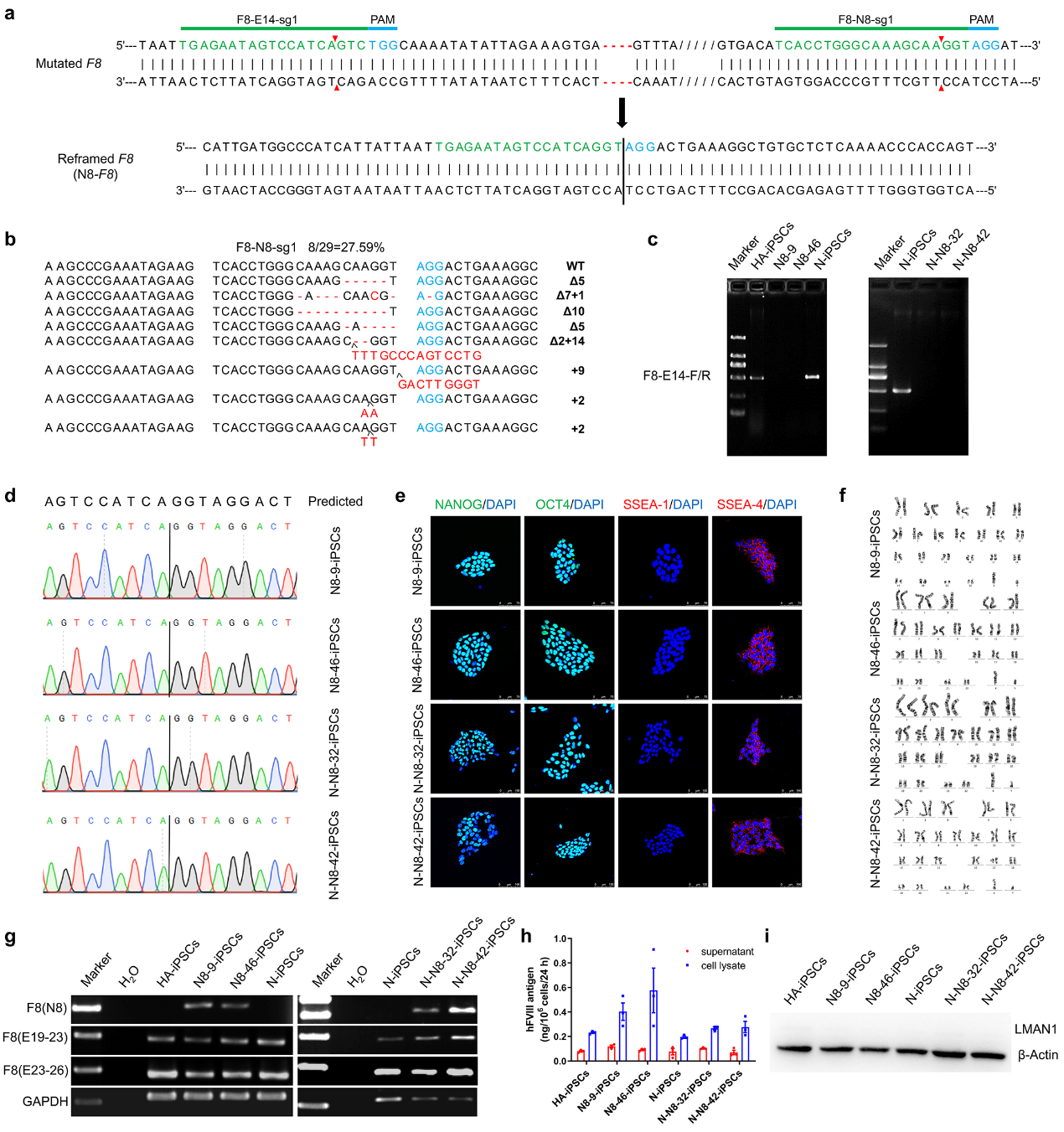


**Figure S4. Generation of iPSCs with 8 N-linked glycosylation sites in B domain of FVIII (N8-iPSCs) mediated by CRISPR/Cas9.**

**a** Schematic representation of CRISPR/Cas9-mediated precise deletion. The red-dotted line indicates the c.3167del CTGA, green letters show the sgRNA sequences, and blue letters are the PAM. The red arrow represents the CRISPR/Cas9 cut sites. Reframed *F8* shows the FVIII-coding sequences with eight N-linked glycosylation sites in the B domain. The vertical line indicates the upstream and downstream connections. **b** Sanger sequencing of the indels induced by F8-N8-sg1 in HEK-293T cells. WT, wild type; △, deletion; +, insertion; ×, times. **c** PCR screening of N8-iPSCs using primers F8-E14-F/R. No PCR product was obtained for the N8-iPSCs. Sizes of the PCR products: N-iPSCs, 502 bp; and HA-iPSCs, 498 bp. **d** The PCR products of N8-iPSCs using primer BUF/BDR were Sanger sequenced. The vertical line indicates the upstream and downstream connections. **e** The N8-iPSC line expressed NANOG, OCT4, and SSEA-4 but not SSEA-1 according to immunofluorescence staining. DAPI was used for nuclear staining. **f** Karyotypes of N8-9-iPSCs and N8-46-iPSCs and **c** N-N8-32-iPSCs and N-N8-42-iPSCs. **g** The transcription of *F8* in iPSCs was analyzed via RT-PCR. The primers in exon 14 and exon 15 (N8), primers in exon 19 and exon 23 (19-23), primers in exon 23 and exon 26 (23-26) were used to detect *F8* transcription, *GAPDH* was a loading control. **h** FVIII antigen of iPSCs was measured via ELISA. Data are represented as the mean ± SEM, n = 3 independent cultures. **i** Western blot of the LMAN1 protein in iPSCs. β-Actin was used as the internal reference.

Figure. S5.


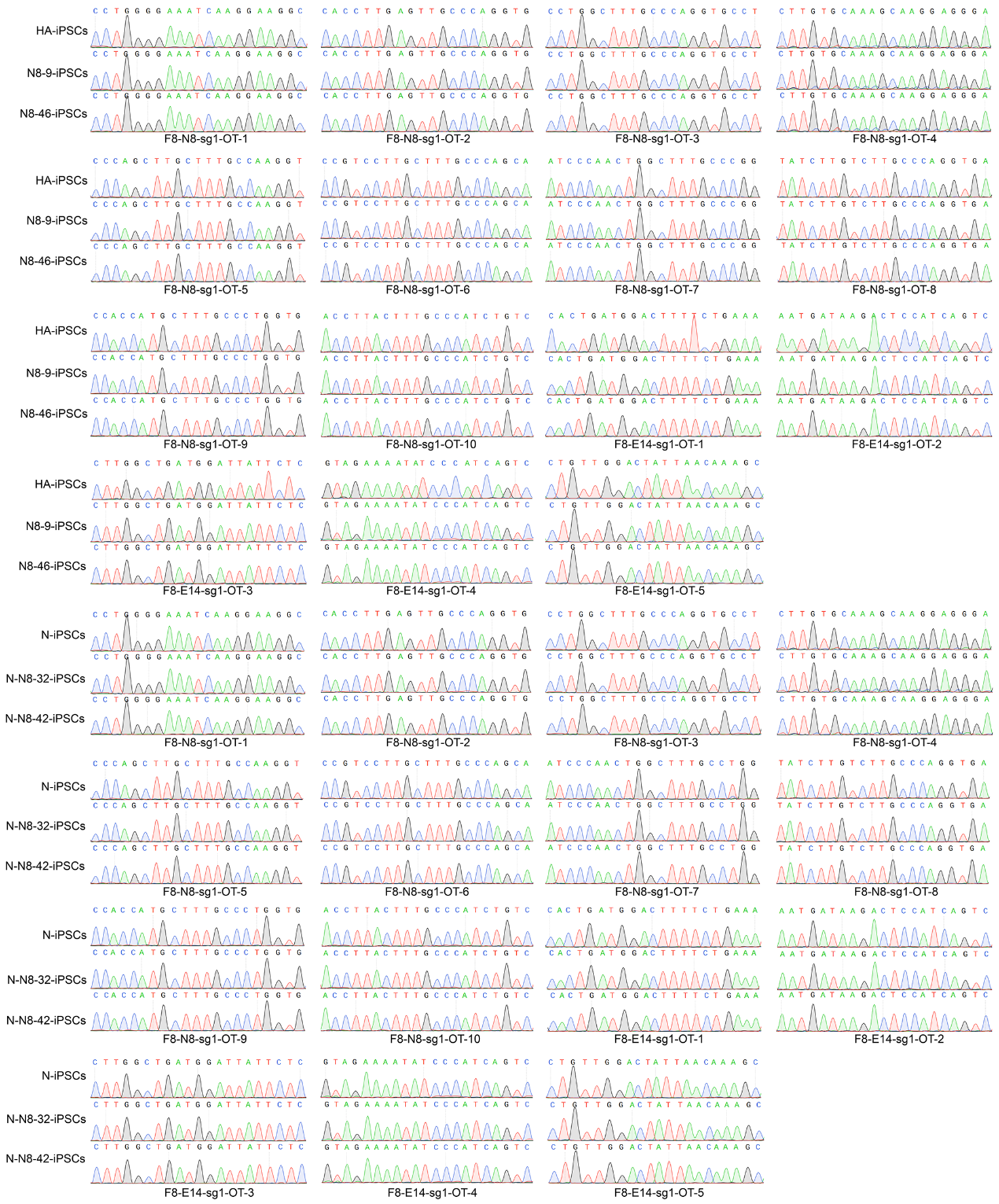


**Figure S5**. **Sanger sequencing of potential off-target sites in N8-iPSCs using F8-N8-sg1 and F8-E14-sg1.**

We analyzed the initial patient cells (HA-iPSCs) and the corrected clones (N8-9-iPSCs and N8-46-iPSCs). And also, we detected the N-iPSCs and the corrected clones (N-N8-32-iPSCs and N-N8-42-iPSCs). No indels were found at the sites.

Figure. S6.


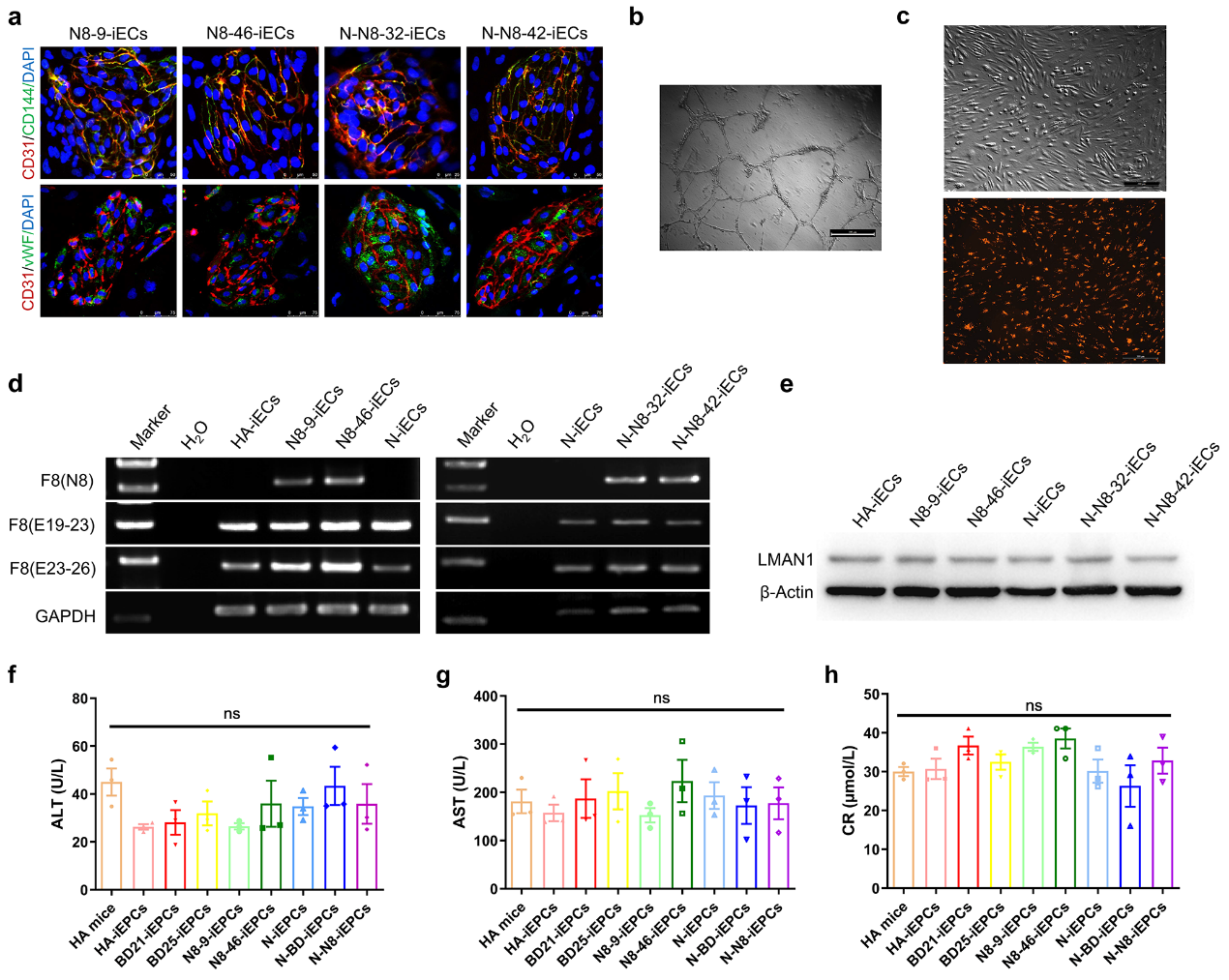


**Figure S6. The FVIII expression was restored in N8-iEPCs derived from N8-iPSCs and liver and kidney function evaluation in HA mice transplanted with EPCs.**

**a** The ECs markers for CD31 (red), CD144 (green) and vWF (green) were visualized using immunofluorescence staining. DAPI was used to mark the nucleus. **b** Angiogenesis assay of ECs. **c** Dil-acLDL endocytosis in ECs. Endocytic Dil-acLDL was visualized as a red fluorescent signal. **d** RT-PCR analysis of *F8* expression in iECs. The primers in exon 14 and exon 15 (N8), primers in exon 19 and exon 23 (19-23), primers in exon 23 and exon 26 (23-26) were used to detect *F8* transcription, *GAPDH* was used as a loading control. **e** Western blot of the LMAN1 protein in iECs. β-Actin was used as the internal reference. Concentrations of **f** alanine aminotransferase (ALT) and **g** aspartate aminotransferase (AST) in HA mice and HA mice transplanted with EPCs. **h** Creatinine (CR) concentration.

Tables. S1 to S4

Table. S1.

| **Primer name** | **Sequence (5’ to 3’)** | **Used for the experiment of** |
| --- | --- | --- |
| F8-E14-sg1-F | CACCGTGAGAATAGTCCATCAGTC | Construction of F8-E14-sgRNA1 |
| F8-E14-sg1-R | AAACGACTGATGGACTATTCTCAC |  |
| F8-BDU-sg1-F | CACCGCTAGGGTGTCTTGAATTCT | Construction of F8-BDU-sgRNA1 |
| F8-BDU-sg1-R | AAACAGAATTCAAGACACCCTAGC |  |
| F8-BDD-sg4-F | CACCGATGGCGTTTCAAGACTGGT | Construction of F8-BDD-sgRNA4 |
| F8-BDD-sg4-R | AAACACCAGTCTTGAAACGCCATC |  |
| F8-N8-sg1-F | CACCGTCACCTGGGCAAAGCAAGGT | Construction of F8-N8-sgRNA1 |
| F8-N8-sg1-R | AAACACCTTGCTTTGCCCAGGTGAC |  |
| F8-E14-F | GGTGGACCTCTGAGCTTGAGTGA | PCR for reframed clones screening |
| F8-E14-R | GCATCTTAAAGAACGACATATCTGGAT |  |
| GAPDH-F | GGGGAGCCAAAAGGGTCATCATCT | RT-PCR for GAPDH |
| GAPDH-R | GACGCCTGCTTCACCACCTTCTTG |  |
| F8-RT-E19F | GCTGGGATGAGCACACTTTT | RT-PCR for F8(E19-23) |
| F8-RT-E23R | TCAACTCCATGCGAAGAGTG |  |
| F8-RT-E23F | CACTCTTCGCATGGAGTTGA | RT-PCR for F8(E23-26) |
| F8-RT-E26R | GGGGGTGAATTCGAAGGTAG |  |
| F8-BDD-ssODN | TAAAAACAATGCCATTGAACCAAGAAGCTTCTCCCAGAATCCACCAGTCTTGAAACGCCATCAACGGGAAATAACTCGTA | ssODN for gene targeting |
| BUF | CATGACCGCCTTACTGAAGGT | PCR for reframed clones screening and cleavage efficiency detecting of F8-BDU-sg1 |
| BUR | CATGACCGCCTTACTGAAGGT |  |
| BDF | TCTCCCGAAACCAGACTTGC | PCR for reframed clones screening and cleavage efficiency detecting of F8-BDD-sg4 and F8-N8-sg1 |
| BDR | AATCCCAGAGCCTCTCCACT |  |
| F8-RT-BDF | ACTCACCCTATTCCCATTCTCA | RT-PCR for F8(BD) and F8(N8) |
| F8-RT-BDR | TGAGTAAAGGAGCCATCAGTAAAT |  |
| F8-RT-N8-F | TATCGTCAACAGAGAGTGGTAGGT |  |

**Primers used in genotyping and RT-PCR**

Table. S2.

| **sequence** | **mismatches** | **locus** |
| --- | --- | --- |
| CCAGGACTTCAAGACACCCTGGC | 3MMs[4:7:21] with F8-BDU-sg1 | chr11:9312306 |
| CCAATAATTCAAGACTCACTAGC | 3MMs[5:16:18] with F8-BDU-sg1 | chr12:99607601 |
| GATAGGTGGTCTTGAATTCTTGG | 3MMs[2:7:8] with F8-BDU-sg1 | chr14:20348081 |
| CCCAGACTTTGAGACACCCTAGC | 3MMs[7:10:11] with F8-BDU-sg1 | chr14:39474980 |
| CCCAGCCTTCAAGACACCCTGGC | 3MMs[6:7:21] with F8-BDU-sg1 | chr14:68785575 |
| CCAAGAATTCAAGACAGCCTGGC | 2MMs[17:21]with F8-BDU-sg1 | chr17:56882309 |
| GCTAGGGTGGCTTGAAGACTGGG | 3MMs[10:17:18] with F8-BDU-sg1 | chr2:170737907 |
| CCTAGAAACCATGACACCCTAGC | 3MMs[8:9:12] with F8-BDU-sg1 | chr5:3971855 |
| AATGGCGTTTCAAAACTGGCTGG | 3MMs[1:14:20] with F8-BDD-sg4 | chr11:34259697 |
| CCCACCATTCTTGAAACTCCTTC | 3MMs[8:18:21] with F8-BDD-sg4 | chr14:45560126 |
| CCTACCAGTCTTGACATGCCAAC | 3MMs[15:17:22] with F8-BDD-sg4 | chr6:117200412 |

**Potential off-target sites of F8-BDU-sg1 and F8-BDD-sg4 predicted by the CHOPCHOP**

The off-target sites were predicted by the CHOPCHOP. The mismatch is indicated by colored letters.

Table. S3.

**Potential off-target sites of F8-N8-sg1 and F8-E14-sg1 predicted by the CHOPCHOP**

| **sequence** | **mismatches** | **locus** |
| --- | --- | --- |
| CCTTCCTTGATTTCCCCAGGTGA | 3MMs[4:10:14] with F8-N8-sg1 | chr1:87182120 |
| TCACCTGGGCAACTCAAGGTGGG | 2MMs[13:14] with F8-N8-sg1 | chr10:47021905 |
| GCACCTGGGCAAAGCCAGGTTGG | 2MMs[1:16] with F8-N8-sg1 | chr15:88880078 |
| CCCTCCTTGCTTTGCACAAGTGA | 3MMs[4:16:19] with F8-N8-sg1 | chr16:15804094 |
| GCACCTTGGCAAAGCAAGCTGGG | 3MMs[1:7:19] with F8-N8-sg1 | chr16:88768918 |
| TCTGCTGGGCAAAGCAAGGACGG | 3MMs[3:4:20] with F8-N8-sg1 | chr22:32818272 |
| TCACCCGGGCAAAGCCAGTTGGG | 3MMs[6:16:19] with F8-N8-sg1 | chr22:44449474 |
| TCACCTGGGCAAGACAAGATAGG | 3MMs[13:14:19] with F8-N8-sg1 | chr5:63831583 |
| GCACCAGGGCAAAGCATGGTGGG | 3MMs[1:6:17] with F8-N8-sg1 | chr7:138113869 |
| TCAGATGGGCAAAGTAAGGTAGG | 3MMs[4:5:15] with F8-N8-sg1 | chr8:31631264 |
| TTCAGAAAAGTCCATCAGTGCAG | 3MMs[3:8:20] with F8-E14-sg1 | chr6:116338171 |
| ATGATAAGACTCCATCAGTCTGG | 4MMs[1:5:8:10] with F8-E14-sg1 | chr3:121903943 |
| TGGAGAATAATCCATCAGCCAAG | 3MMs[2:10:19] with F8-E14-sg1 | chr1:175915498 |
| TAGAAAATATCCCATCAGTCCAG | 4MMs[2:5:10:11] with F8-E14-sg1 | chr5:13817688 |
| TTGTTAATAGTCCAACAGTTGGG | 4MMs[4:5:15:20] with F8-E14-sg1 | chr2:198572468 |

The off-target sites were predicted by the CHOPCHOP. The mismatch is indicated by colored letters.

Table. S4.

**Primers used in CRISPR/Cas9 off-target analysis**

| **Site** | **Forward primer** | **Reverse primer** | **Expected product size, bp** |
| --- | --- | --- | --- |
| F8- BDU-sg1-OT-1 | AGTTTCACACTAACCTTCAACCCTA | CAATTATTCTTATGATGCTGTCCC | 476 |
| F8- BDU-sg1-OT-2 | TTTTTTCCCCCATGACTCATATA | TCCTCAGTTACCAGCCTAAAATCT | 555 |
| F8- BDU-sg1-OT-3 | CCAGCAATCCTCCCACCTAA | ACAAATCTGGAAAACACAAGTGG | 319 |
| F8- BDU-sg1-OT-4 | TCTTCTAAACTTTGTCACTTCCCA | CCAATTCATTTCACAAAGCCA | 514 |
| F8- BDU-sg1-OT-5 | TGCCTCTTCGGTAACCAGTG | CCCATTATTTCTCCAGGATGTTAG | 571 |
| F8- BDU-sg1-OT-6 | TAAACAGTTGGACATAAAGGGGTA | TCGGCTCACAGCAACCTCT | 458 |
| F8- BDU-sg1-OT-7 | CATTTGGTTGATCTTCCTTTATTTC | CTCTCAGGACACTCCCTCACC | 475 |
| F8- BDU-sg1-OT-8 | ATGGGAAGCATCCAGTTGAG | CTTTGGGAGGTTGAGGTGAA | 479 |
| F8- BDD-sg4-OT-1 | CAGGAATAAAGCAGGGAAGATAA | GAGGCTAAGGTAGGAGGATTGC | 588 |
| F8- BDD-sg4-OT-2 | CCGCTCTATCTATCAGGAACACC | TGGAAACAGCATCTTCAACAAC | 447 |
| F8- BDD-sg4-OT-3 | GTGCTAAATCTTCCTATCCCTCTT | TAAAAAGCCCTAATGAGAAAGTGT | 374 |
| F8-N8-sg1-OT-1 | CCTTGTGCCCTTGCTGAAC | TCAGGTCCATTCTCATCCTACAT | 588 |
| F8-N8-sg1-OT-2 | GGGGAAGGGGATTTCAGTTT | GGGTTTATGCCCTGTCTTTGA | 521 |
| F8-N8-sg1-OT-3 | GGGAAGGACACTTTGGAATCTC | ACTTTAGCCCCAGCCCGT | 553 |
| F8-N8-sg1-OT-4 | CTGGTTACGAGCAGAAGGACT | CCACCTCCCTGGTTCACG | 523 |
| F8-N8-sg1-OT-5 | CAGAGCGAGCAGCCGAAC | CCAAGATACCTCATTATGCGTGT | 608 |
| F8-N8-sg1-OT-6 | ACATCACCGTCCTACCTAATCAT | CAGAGGACTCAAAGGGAAGG | 558 |
| F8-N8-sg1-OT-7 | CCCTGTCCATCCTGGTAAGTC | GAAAGGAGGGAGCATTTAATAGAC | 394 |
| F8-N8-sg1-OT-8 | GAACCAGGTAGGAAGATGCCA | ACAGGAGTTAGGTGGGTACAGG | 635 |
| F8-N8-sg1-OT-9 | ACACCAGCCTGACTCCTCTTAG | TGCCTTCACCGAAGTCACAA | 457 |
| F8-N8-sg1-OT-10 | TCAGTGCCCTCCTTGACCTA | TGACCCAGCCATCCCATTA | 578 |
| F8-E14-sg1-OT-1 | TTTGGTTGCTCAAAACTTGG | AGAAAGCCTGAAACAGTGCC | 416 |
| F8-E14-sg1-OT-2 | GCTGTCTTGGGAACTTTGCT | CCCTGAGGACCACCTTGTGA | 419 |
| F8-E14-sg1-OT-3 | GAACTTTCTAAACAAAACACCCT | ATGCTGGCAATCCCAATT | 552 |
| F8-E14-sg1-OT-4 | TCGGGAAATGAGGATGAAT | AAAAATAGGCTGTACTTAAATGAGA | 499 |
| F8-E14-sg1-OT-5 | TCAGGTGCCTGCTCCTATT | ATTCCGAATCCTCAATCTTTA | 510 |
